# Comparative Evaluation of Alveolar Nerve Block with 2% Lidocaine–Epinephrine and 4% Articaine–Epinephrine Buccal Infiltration in Mandibular Premolar and Molar Region in Children: A Double-Blind, Randomized Trial

**DOI:** 10.3390/children12020215

**Published:** 2025-02-12

**Authors:** Jelena Komsic, Sanja Vujkov, Isidora Neskovic, Duska Blagojevic, Ana Tadic, Bojan Petrovic, Branislav Bajkin

**Affiliations:** 1 Department of Dentistry, Medical Faculty, University of Novi Sad, 21000 Novi Sad, Serbia; sanja.vujkov@mf.uns.ac.rs (S.V.); isidora.neskovic@mf.uns.ac.rs (I.N.); duska.blagojevic@mf.uns.ac.rs (D.B.); ana.tadic@mf.uns.ac.rs (A.T.); bojan.petrovic@mf.uns.ac.rs (B.P.); branislav.bajkin@mf.uns.ac.rs (B.B.); 2 Dentistry Clinic of Vojvodina, 21102 Novi Sad, Serbia

**Keywords:** dental anesthesia, inferior alveolar nerve, buccal administration, articaine, lidocaine, pediatrics

## Abstract

**Background/Objectives**: Effective pain control in pediatric dentistry combines behavior management, local anesthesia, and follow-up care. This study compared the efficacy of inferior alveolar nerve block (IANB) with 2% lidocaine and epinephrine versus buccal infiltration (BI) with 4% articaine and epinephrine in treating primary molars, permanent premolars, and molars in children. **Methods**: Sixty children aged 5–18 years were randomly assigned to two groups in a double-blind study. One group received 1.7 mL BI with 4% articaine, and the other 1.8 mL IANB with 2% lidocaine for dental treatment. Pain was assessed using the self-reported Visual Analog Scale (VAS) and Wong–Baker Faces Pain Rating Scale (W-BFRS), while anesthesia effectiveness and behavior were evaluated using the Frankl Behavior Rating Scale (FBRS) and vitality tests. **Results**: The articaine group reported significantly lower pain scores on all scales (VAS: 1.23 ± 2.01; FBRS: 0.47 ± 0.57; W-BFRS: 1.33 ± 2.04) than the lidocaine group (VAS: 3.17 ± 1.64; FBRS: 1.26 ± 0.45; W-BFRS: 3.17 ± 1.64). Articaine also outperformed lidocaine in secondary measures, with higher positive responses on the questionnaires (articaine: 8.37 ± 1.61 vs. lidocaine: 5.27 ± 1.41, *p* < 0.001). **Conclusions**: Buccal infiltration with 4% articaine is more effective than 2% lidocaine administered via IANB for invasive dental procedures in children, providing superior pain control and positive patient responses.

## 1. Introduction

Optimal anesthesia is a pivotal element in ensuring the success of dentoalveolar interventions, particularly in pediatric dentistry where managing pain holds significant importance [1]. Effective pain control in pediatric dentistry requires a combination of behavioral management, local anesthesia, and follow-up care tailored to the child’s age, temperament, and the specific dental procedure. The goal is to minimize pain, reduce anxiety, and foster a positive dental experience that encourages continued regular dental care in the future [2]. Pain during childhood dental treatments can lead to long-term fear, anxiety, and behavioral issues [2,3].

Effective pain control in mandibular dental procedures requires blocking the inferior alveolar, buccal, and lingual nerves. The inferior alveolar nerve enters the mandibular canal via the mandibular foramen, protected by the lingula, and provides sensory innervation to mandibular teeth, bone, and soft tissues [4,5]. The lingual nerve innervates the lingual soft tissues, mouth floor, and anterior two-thirds of the tongue, while the buccal nerve supplies the vestibular soft tissues [6]. The Spix technique, which anesthetizes the inferior alveolar and lingual nerves by targeting the lingula, is the most common approach. Landmarks include the anterior ramus border, occlusal plane, and pterygomandibular raphe. In adults, the mandibular foramen is about 10 mm above the occlusal plane, while in children, it is closer to the occlusal plane and anterior ramus, shifting upward with mandibular growth [6,7]. Despite its use, the Spix technique is often uncomfortable, inconsistent, and prone to complications such as hematoma and intravascular injection. Alternative techniques like Gow-Gates and Vazirani–Akinosi have been introduced to improve outcomes, but remain suboptimal. Further exploration of better methods, especially for children, is necessary [8].

Solutions of local anesthetics used in dental medicine typically contain a local anesthetic to which a vasoconstrictor is frequently added. The most commonly used local anesthetics belong to the amide group, characterized by a lipophilic aromatic ring and a hydrophilic amino group connected by an amid-type central chain. The most common local anesthetics in practice are lidocaine and mepivacaine, but articaine and bupivacaine are also becoming increasingly popular. As for the vasoconstrictors added, adrenaline (epinephrine), noradrenaline (norepinephrine), levonordefrin, and felipressin are primarily used [6].

Traditionally, the Inferior Alveolar Nerve Block (IANB) has been the preferred anesthetic technique, but the advent of articaine, a newer local anesthetic with superior tissue diffusion properties, has sparked interest in alternative approaches [2]. Following the global approval of articaine, researchers have turned their attention to the comparative efficacy of Buccal Articaine Infiltration (BI) and IANB in the mandibular posterior teeth. Several studies, predominantly conducted on healthy volunteers, have consistently reported comparable results between the two techniques, prompting a reevaluation of the standard IANB approach [3,9,10]. This study delves into the effectiveness of local infiltration anesthesia using 4% articaine specifically in the analgesia of deciduous and permanent molars in children aged 5–18 years and mandibular premolars in children aged 10–18 years. The primary objective is to ensure a painless and effective implementation of dental procedures in children, considering the associated benefits of reduced stress, increased success rates, and lower complication rates associated with infiltration anesthesia [10].

The main aim of this study is to establish the feasibility of conducting a double-blind randomized trial, scrutinizing the clinical differences between the standard IANB using 2% lidocaine with 1:80,000 epinephrine and Buccal Infiltration (BI) injection using 4% articaine with 1:100,000 epinephrine. The primary outcome revolves around discerning potential disparities in pain experienced during treatment between the two techniques, with a secondary focus on evaluating the pain experienced during injection.

## 2. Materials and Methods

This study was approved by the Ethics Committee of the Dental Clinic of Vojvodina, Medical faculty, University of Novi Sad (Number: 01-18/12-2020), and recorded and approved on ClinicalTrials.gov PRS (ID:NCT05423392). The study was conducted at the Department of Preventive and Pediatric dentistry of the Dental Clinic of Vojvodina from 1 January 2022 to 30 April 2022.

### 2.1. Design and Inclusion Critera

The research included 60 participants aged 5–18. Using G*Power Version 3.1.9.6 analysis, the sample size determined for the study was at least 34 participants divided into two groups. In this study, 92 patients were assessed for eligibility. Excluded were 32 patients: 27 patients did not meet inclusion criteria, 3 patients declined to participate, and 2 patients were not cooperative. Undergoing randomization were 60 patients.

The procedure was explained to the parents and the child in the language best understood by them. Participants (or parents/guardians) who were included in the clinical study signed a consent form to participate in the clinical study, but did not know which anesthetic would be received.

The patients were allocated to two groups using a random numbers table in Excel 2013 (Microsoft support, RAND function). One group of 30 participants used the local anesthetic, 4% articaine epinephrine. The second group of 30 participants were provided with the local anesthetic, 2% lidocaine epinephrine. For analysis and data collection in each group, subgroups were created depending on the age of the participants: 5–9 years, 10–13 years, and 14–18 years.

The study was conducted according to the ethical principles of Good Clinical Practice (GCP), in accordance with the latest revision of the Declaration of Helsinki. The patients’ (or parents’/guardians’) written Informed Consent implied that patients had received full information about research and stressed that they had the right to decide independently to participate, without coercion, external influences, or any harmful consequences if they refused to participate.

Inclusion criteria were as follows.

- Participants with ASA physical status I and II;
- Children over 20 kg of body weight;
- Respondents of both sexes, aged 5–18 years, in need of either conservative rehabilitation or tooth of premolars and/or of deciduous or permanent molars;
- X-rays of the teeth that need to be treated;
- Signed informative consent by patient or parent/guardian.

Exclusion criteria were as follows.

- Difficult cooperation from patients;
- Existence of allergy to local anesthetic;
- Existence of a diagnosed general disease;
- Unsigned informative consent.

### 2.2. Clinical Procedures

As the local anesthesia delivery technique was different for both groups, the operators could not be blinded in the study. The study was double blinded where the participants and the examiners were unaware which anesthetic was applied. The children’s behavior was monitored through all phases of clinical work by direct observation from two dentists (examiners B.P. and S.V.) who were in charge of measuring the effectiveness of the anesthetics, but did not know what type of anesthetic. Only two dentists who administered the local anesthetic (operators, J.K. and I.N.) knew what type of anesthetic was used for the intervention. The dental treatments were tooth extraction, endodontic dental treatment, and conservative tooth restoration of deciduous and permanent mandibular molars in children aged 5–18 years and mandibular premolars in children aged 10–18 years.

The first group followed placement 0.2 mL of 5% lidocaine topical anesthetic (Lidocaine chloride 5% gel Galenika ad, Belgrade, Serbia) according to the manufacturer’s instructions, using a cotton-tipped applicator at the site of the injection on dried mucosa. After a waiting period of 30 s for the topical anesthetic agent to act, local anesthesia was delivered as per the technique chosen [2]. For all injections, a self-aspirating metal syringe was used (Sopira, Kulzer GmbH Leipziger Straße 2 63450 Hanau, Germany), Syringe Aspirating). The first group for indicated dental treatment received 1.7 mL 4% articaine with 1:100,000 epinephrine (Ubistesin Forte, 3M Deutschland GmbH Carl-Schurz-Straße 1 41453 Neuss Germany, 3M ESPE), buccally in the lateral region of the lower jaw. The anesthetic agent was delivered using needles of 27 gauge and 25 mm length (Denject, Biodent, 446-7 Noijo-ri Jori-Eup Paju-city Guynggi-do Korea(Zip Code: 413-821). On the other side, IANB with 2% lidocaine with 1:80,000 epinephrine 1.8 mL (Lignospan special, Septodont, 127 boulevard Diderot, 75012 Paris, France) was used per the technique mentioned by Malamed in 1997 [2]. The anesthetic agent was delivered using needles of 27 gauge and 35 mm length (Denject, Biodent). The onset of action of the anesthetic agent was checked by the evaluation of subjective and objective symptoms.

### 2.3. Evaluation of Anestethic Efficacy

**Figure 1:**
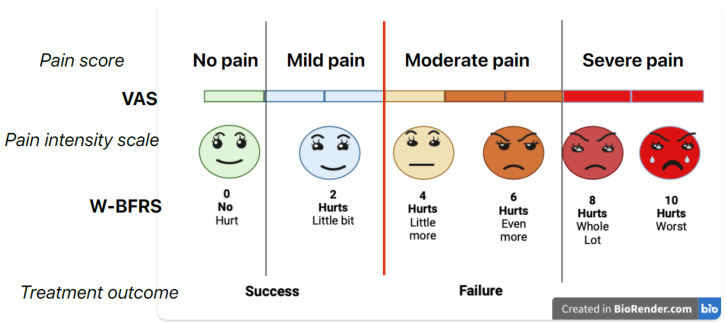
W–BFRS score of 0–2 and VAS of up to 4 was considered as mild pain for the purposes of this study. Mild pain or no pain during the injection and treatment was considered as a success.

The criteria for measuring efficacy were measuring pain during anesthetic injection, 10 min after injection, during and after the intervention. Pain was scored by the patient using the Visual Analog Scale (VAS) and Wong–Baker Pain Rating Scale (W–BFRS) (Figure 1). The children’s behavior was monitored through all phases of clinical work by direct observation from the dentists (examiners B.P and S.V.) using the Frank Behavior Rating Scale (FBRS). The efficacy of anesthesia was measured by the vitality electric test, by determining the growth and development of the roots of permanent mandibular premolars and molars (before intervention) in cases where it was obvious that the tooth was not vital (presence of periapical lesions, root resorptions). The vitality test was not performed preoperatively; the resorption of the roots of the deciduous mandibular molars was determined prior to intervention using X-ray. After that, the examiners filled out the questionnaire based on the answers of the children or parents/guardians.

### 2.4. Method

#### 2.4.1. Study Sample

Overall, 60 children participated in the study; 30 were in the Articaine, and 30 were in the Lidocaine group. In each group, participants were matched by gender, tooth type, and consecutive treatment (the detailed table with sample characteristics is in the Supplementary Material).

#### 2.4.2. Statistical Analysis

To examine the main effect of treatment with two groups (one receiving lidocaine and one receiving articaine) using scores on a scale (i.e., conducting a separate F-test for each pain scale), the analysis is presented below for α = 0.05 (i.e., rejection of the null hypothesis at the *p* = 0.05 level), for the test power of β = 0.80, and for the effect size of 0.5 (partial squared eta = 0.2, i.e., equivalent to the G potency effect). According to these parameters, 34 respondents are enough.

To study the main effect of treatment plus another factor (gender or tooth type), a total of 34 subjects were studied, when two bivalent factors with the same indicators of alpha and beta and the size of the effect (11 for each group) are also needed.

A 2 × 2 ANOVA was applied, with the gender and the treatment group as fixed factors. The second analysis included 2 × 2 ANOVA, as well, with the teeth type and the treatment group as fixed factors, and the third analysis was 3 × 2 ANOVA, with the intervention type and the treatment group as fixed factors. Dependent variables were scores of ratings on four scales, three of which measured the pain: VAS (10-point scale for subjective pain estimation), W–BFRS (subjective estimation of pain on the pictorial scale), and FBRS (estimation by an observer of pain on a three-point scale on five indicators; the summative score ranging from 0 to 10). On these scales, a lower score denotes less pain. The fourth score was a secondary outcome measure, calculated from the questionnaire, where five or more positive answers out of ten indicate positive outcomes of the treatment.

## 3. Results

Table 1 presents participants’ summative demographic characteristics and the perioperative parameters. All statistical tests were non-significant, meaning the articaine and lidocaine groups were properly matched by gender, intervention, and tooth type.

**Figure 2:**
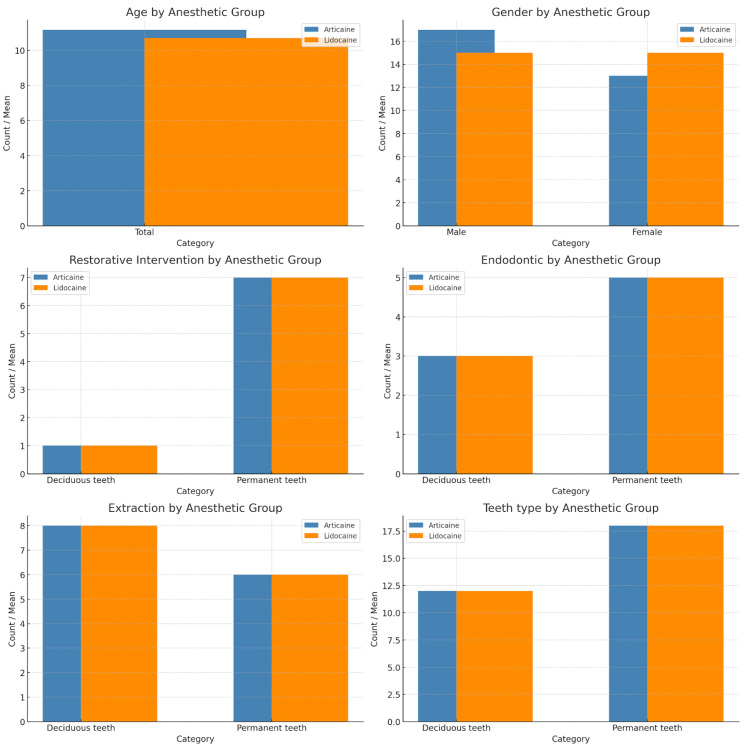
Investigated parameters in the experimental groups.

The applied anesthesia proved to be effective in all cases, meaning that all teeth subjected to vitality tests showed negative results. Boys and girls did not differ by pain level in either of the rating scales (gender main effects were not significant). Concerning the subjective measures of pain, the only significant difference was recorded between the two anesthetic groups, where the articaine group demonstrated lower pain levels on all subjective pain rating scales (*M*_VAS_ = 1.23 ± 2.01; *M*_FBRS_ = 0.47 ± 0.57; *M*_W-BRS_ = 1.33 ± 2.04) compared to the lidocaine group (*M*_VAS_ = 3.17 ± 1.64; *M*_FBRS_ = 1.26 ± 0.45; *M*_W-BRS_ = 3.17 ± 1.64). All *F* tests were significant, and the values are presented in Figure 2 and Table 2.

The articaine group also had better scores on the secondary outcome measure, having, on average, significantly more positive answers on the questionnaire (*M =* 8.37 ± 1.61), compared to the lidocaine group (*M =* 5.27 ± 1.41): *F*(1,56) = 60.81, *p* < 0.001.

All differences between the two treatment groups were significant, as in the previous analysis. Concerning the teeth type, as expected, both anesthetics showed better performance (had better scores on every pain scale) on participants who received interventions on deciduous teeth. There was no significant interaction between treatment groups and teeth type. This means that articaine has overall better performance compared to lidocaine regardless of the teeth type.

**Figure 3:**
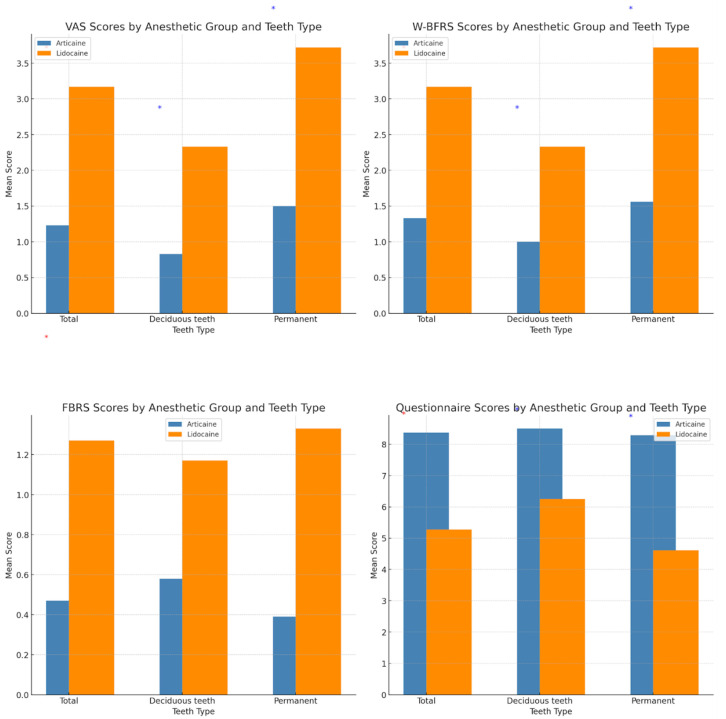
VAS—visual analog scale; W–BFRS—Wong–Baker Pain Rating Scale; FBRS—Frank Behavior Rating Scale across the experimental group.* By *t* test.

The third analysis showed only a significant effect for the treatment group, as shown in the previous two analyses. Both lidocaine and articaine showed equally successful effects within the group regardless of the intervention type. There were no differences between the interventions concerning the evaluation of pain (Figure 3 and Table 3).

## 4. Discussion

This study aimed to evaluate the comparative efficacy of inferior alveolar nerve block (IANB) with 2% lidocaine and epinephrine versus buccal infiltration (BI) with 4% articaine and epinephrine for dental treatments in children aged 5 to 18 years. The findings demonstrated that articaine BI consistently resulted in lower pain scores across all scales (VAS, W–BFRS, FBRS) compared to lidocaine IANB. Additionally, articaine BI showed a higher proportion of positive responses on post-treatment questionnaires. These results suggest that BI with articaine may be a superior option for managing pain in pediatric dental procedures, aligning with the growing body of evidence supporting its efficacy and safety.

A comparison was made between the mean values on various pain scales for the two groups of anesthetics, namely articaine (N = 30) and lidocaine (N = 30), and also between the male and female cohorts. Although the hypothesis focused on overall analgesic efficacy during treatment, pain experienced during injection was also assessed, as it is a critical factor in pediatric patient comfort. Injection-related discomfort can contribute to procedural anxiety, influencing cooperation and the overall dental experience. Evaluating both stages (injection and treatment) provides a more comprehensive understanding of the patient’s response to different anesthetic techniques. The assessment of pain perception and behavior during the dental procedures involved the utilization of the visual analog scale (VAS), Wong–Baker Pain Rating Scale (W–BFRS), Frank Behavior Rating Scale (FBRS), and a questionnaire. Significant variances were evident across all pain scales between the two anesthetic groups. Patients administered with articaine exhibited notably lower pain ratings in contrast to those receiving lidocaine, as denoted by the decreased mean values on the VAS, W–BFRS, and FBRS (*p* < 0.05 for all comparisons). These outcomes propose that articaine may offer more effective pain alleviation than lidocaine in the context of dental procedures. Furthermore, an exploration into gender disparities revealed no consequential interaction effects between gender and anesthetic group on any of the pain scales (*p* > 0.05). These results affirm that the effectiveness of articaine and lidocaine in pain management remains consistent regardless of gender, advocating for the applicability of both anesthetics among male and female patients undergoing dental treatments. Our results align with previous research comparing the effectiveness of articaine and lidocaine in pain management during dental procedures in pediatric patients. A study demonstrated that articaine hydrochloride 4% with epinephrine 1:100,000 was both efficacious and safe for the treatment of children aged 3 to 6 years [11]. The findings featured in the International Journal of Paediatric Dentistry revealed that articaine outperformed lidocaine in achieving successful anesthesia in pediatric dentistry [12]. Conversely, a different randomized controlled trial concluded that there was no notable disparity in pain and anxiety levels when comparing lidocaine to articaine in children undergoing extensive dental work under general anesthesia [13]. Nonetheless, it is crucial to emphasize the prudent use of articaine in pediatric patients, particularly those under 4 years of age, adhering to established guidelines, given that certain practitioners may lack awareness of the specific recommendations for pediatric application [11]. To comprehensively evaluate the impact of articaine and lidocaine on various demographic and procedural factors, we conducted an analysis that included additional variables such as age, gender distribution, and types of dental procedures. The purpose of these analyses was to elucidate any potential relationships between these factors and the effectiveness of the two anesthetic agents in pain management during dental treatments. Initially, our findings indicated no statistically significant variances in age between patients who received articaine and those who received lidocaine (*p* = 0.635), suggesting that age did not play a role in the selection of anesthetic agents within our study cohort. Similarly, the distribution of gender did not show any substantial differences between the two groups (*p* = 0.605), demonstrating a relatively equal representation of male and female participants in both cohorts. With respect to the types of dental procedures, our results displayed similar distributions between the articaine and lidocaine cohorts for restorative treatments, endodontic procedures, and extractions. There were no notable differences observed in the distribution of deciduous versus permanent teeth between the two groups, indicating a comparable utilization of articaine and lidocaine in various dental interventions. Moreover, the statistical analyses conducted using 2 × 2 and 3 × 2 ANOVA models did not reveal any significant interactions between the treatment groups (articaine vs. lidocaine) and the demographic or procedural variables that were examined. These outcomes suggest that the efficacy of articaine and lidocaine in pain management during dental procedures remained constant across diverse patient demographics and types of dental treatments. Our results suggest that factors such as age, gender, and the nature of dental intervention do not have a substantial impact on the selection or effectiveness of anesthetic agents in pain management during dental procedures. These findings offer valuable insights into the clinical considerations associated with the use of articaine and lidocaine in varied patient populations and underscore the significance of personalized anesthesia approaches tailored to individual patient requirements.

Pain control during dental treatment is an essential aspect of pediatric dentistry [14]. Studies in the literature have shown that there is a strong relationship between pain and behavior-related problems in dentistry [10,15,16]. Pain management in pediatric dentistry is crucial not only for the comfort of the child, but also for their overall experience and future behavior related to dental care.

Previously, it has been postulated that the drawbacks associated with employing terminal anesthesia, as opposed to alveolar block, in the field of pediatric dentistry encompasses a heightened incidence of failed anesthesia, insufficiency in anesthesia due to the formation of blood clots, and the necessity for supplementary anesthesia methods in cases involving pre-existing abscess or active infection. Furthermore, terminal anesthesia has the potential to lead to incomplete anesthesia of the mandibular teeth, thereby potentially necessitating further injections to attain satisfactory anesthesia. Moreover, terminal anesthesia may fail to anesthetize the base of the oral cavity or the front two-thirds of the tongue, which could be indispensable for specific dental interventions. Lastly, terminal anesthesia may prove inadequate for more intricate dental procedures like extractions or surgical interventions, necessitating alveolar block anesthesia for optimal pain management.

Appropriate management of dental pain in children prevents development of fear, anxiety, and behavioral problems during treatment [14,15,16,17]. Subjective measures, such as patient-reported outcomes, play a crucial role in assessing the overall experience of dental procedures for children. These measures provide valuable insights into the subjective feelings, perceptions, and satisfaction levels of pediatric patients and their caregivers. Thus, to achieve the aim of this study, the process of pain assessment included asking the child directly the question, “How much does it hurt?” using the pain rating scales VAS, W–BFRS, and FBRS. The VAS is one of the most widely used pain assessment scales in acute pain research. One of the strengths of using a VAS scale as a self-report measure is the range of choices available to the subjects for describing their perceptions [14,15,16,17,18]. In this study, a standard transcript was used to explain VAS to the child. The W–BFRS scale was chosen because it has adequate psychometric properties, and it is easy and quick to use. A systematic review [15,16,17,18,19] identified W–BFRS as one of the scales that has undergone extensive psychometric testing and been used in the assessment of both acute and disease-related pain in children. The FBRS scale was chosen as the behavioral observation method in accordance with previous literature [14,15,16,17,18]. The observed differences in pain levels between articaine and lidocaine in pediatric patients can have several practical implications for dental practitioners in clinical settings. Articaine has been shown in some studies to have a faster onset of action compared to lidocaine. This can be advantageous in pediatric dental settings where quick and effective pain relief is essential, particularly for procedures involving highly sensitive areas or anxious patients. Pediatric dentists are trained in behavioral management techniques to help children remain calm and cooperative during treatment. This may involve distraction techniques, positive reinforcement, and clear communication to alleviate fear and anxiety. By effectively managing pain during dental treatment, pediatric dentists can help ensure a positive experience for children, promote good oral health habits, and prevent long-term behavioral issues related to dental care.

By contrasting data from men and women, the study can investigate whether gender influences the results. Even in the absence of notable distinctions, this unfavorable discovery is still important from a scientific standpoint and adds to the corpus of knowledge [9,20].

The study provides valuable evidence on the comparative effectiveness and safety of articaine in pediatric patients. Minimizing anxiety and fear, the study contributes to efforts aimed at reducing anxiety and promoting a positive dental experience for children. Age-appropriate anesthesia techniques, including the selection of local anesthetics, contribute to building trust and rapport between pediatric patients and their dentists. By choosing the most suitable anesthesia technique based on the child’s age, medical history, and individual needs, dentists can enhance communication, alleviate fear, and establish a positive dentist–patient relationship. This, in turn, promotes better cooperation during dental visits and improves long-term oral health outcomes. Optimal anesthesia, particularly with articaine, contributes to building a strong and positive dentist–patient relationship in pediatric dentistry by reducing pain and discomfort, enhancing comfort and safety, improving communication and engagement, facilitating positive memory formation, and promoting long-term oral health benefits. By prioritizing patient-centered care and establishing trust and comfort during dental procedures in childhood, dentists lay the foundation for a lifetime of positive dental experiences and optimal oral health outcomes for their young patients. Further research, including randomized controlled trials with larger sample sizes and standardized methodologies, would be valuable to confirm and expand upon these findings and to explore the underlying mechanisms contributing to patient preferences for articaine over lidocaine in dental anesthesia. Articaine is not recommended for use in children below 4 years of age due to limited evidence of its safety in this age group [15,19,21,22,23]. Therefore, in this study, children in the 5- to 18-year age group were included.

Several limitations must be acknowledged. The first one is related to the sample size: While sufficient for statistical analysis, the relatively small sample size may limit the generalizability of the findings. Future studies should include larger cohorts to validate these results. The broad age range (5 to 18 years) introduces variability in pain perception and reporting abilities. Stratifying the sample into narrower age groups may provide more nuanced insights into age-related differences in anesthetic efficacy. The study’s double-blind design ensured participant and evaluator blinding, but did not include operator blinding due to the inherent differences in technique. This may introduce subtle biases despite efforts to standardize procedures. Different dental procedures were included, each with varying levels of invasiveness and tissue involvement. While efforts were made to standardize pain assessment, these procedural differences may influence outcomes.

Further research in this area is essential. Considering our primary intention to identify a technique that will effectively anesthetize pediatric patients in the least invasive manner, a comparison should be made with new alternative local anesthesia techniques, such as the retromolar triangle anesthesia technique. This technique is reported to be sufficiently effective and significantly reduces the incidence of complications associated with conduction anesthesia [24].

## 5. Conclusions

Buccal infiltration with 4% articaine was demonstrated to be an effective method for dental treatment in children. Pain experienced during injections was significantly lower with buccal infiltration using 4% articaine compared to inferior alveolar nerve block (IANB) with 2% lidocaine. Additionally, buccal infiltration with 4% articaine received a higher proportion of positive responses in the post-treatment questionnaire than IANB with 2% lidocaine. These findings suggest that invasive dental procedures on mandibular teeth can be successfully completed in children using buccal infiltration with 4% articaine. Further clinical trials with larger sample sizes are recommended to confirm these results and enhance the generalizability of the findings.

## Figures and Tables

**Table 1:** Patients’ demographic and perioperative parameters.

Variable	Articaine (*N* = 30)	Lidocaine (*N* = 30)	Total	*p* Value
Age	11.17 ± 3.78	10.7 ± 3.79		0.635 *
Gender				0.605 ^†^
Male	17	15	32	
Female	13	15	28	
Restorative intervention (N = 16)				
Deciduous teeth	1	1	2	
Permanent teeth	7	7	14	
Endodontic (N = 16)				
Deciduous teeth	3	3	6	
Permanent teeth	5	5	10	
Extraction (N = 28)				
Deciduous teeth	8	8	16	
Permanent teeth	6	6	12	
Teeth type	6	6	12	
Deciduous teeth	12	12	24	
Permanent teeth	18	18	36	

Data are presented as mean ± standard deviation or number; * By *t* test; ^†^ By *χ*^2^ test.

**Table 2:** Mean values on different pain scales by anesthetic groups and by gender, with *F* tests and significance tests.

	Variable	Articaine (*N* = 30)	Lidocaine (*N* = 30)	Total	*F* Test		*p* Value
VAS	Total	1.23 ± 2.01	3.17 ± 1.64		Group	16.50	<0.001
	Male	1.41 ± 2.62	3.27 ± 1.79	2.28 ± 2.43	Gender	0.402	0.529
	Female	1.00 ± 0.71	3.07 ± 1.53	2.11 ± 1.59	Gender × Group	0.048	0.827
W–BFRS	Total	1.33 ± 2.04	3.17 ± 1.64		Group	14.67	<0.001
	Male	1.53 ± 2.63	3.27 ± 1.79	2.34 ± 2.40	Gender	0.450	0.505
	Female	1.08 ± 0.86	3.07 ± 1.53	2.14 ± 1.60	Gender × Group	0.067	0.796
FBRS	Total	0.47 ± 0.57	1.26 ± 0.45		Group	35.11	<0.001
	Male	0.47 ± 0.62	1.20 ± 0.41	0.81 ± 0.64	Gender	0.211	0.647
	Female	0.46 ± 0.52	1.33 ± 0.49	0.93 ± 0.66	Gender × Group	0.278	0.600
Questionnaire	Total	8.37 ± 1.61	5.27 ± 1.41		Group	60.81	<0.001
	Male	8.29 ± 1.99	5.33 ± 1.54	6.90 ± 2.31	Gender	0.002	0.966
	Female	8.46 ± 0.97	5.20 ± 1.32	6.71 ± 2.02	Gender × Group	0.142	0.708
Root development	Unfinished	1	1				
	Finished	17	17				
Resorption of the roots	Resorbed	11	10				

Notes: Data are presented as mean ± standard deviation or as counts; VAS—visual analog scale; W–BFRS—Wong–Baker Pain Rating Scale; FBRS—Frank Behavior Rating Scale; significant differences are bolded.

**Table 3:** Mean values on different pain scales by anesthetic groups and by teeth type, with *F* tests and significance tests.

	Variable	Articaine (*N* = 30)	Lidocaine (*N* = 30)	Total	*F* Test		*p* Value
**VAS**	**Total**	**1.23 ± 2.01**	**3.17 ± 1.64**		**Treatment group**	**15.65**	**<0.001**
	*Deciduous teeth*	0.83 ± 0.58	2.33 ± 0.65	1.58 ± 0.97	Teeth type	4.78	**0.033**
	*Permanent*	1.50 ± 2.55	3.72 ± 1.87	2.61 ± 2.48	Teeth type × Group	0.589	**0.446**
**W–BFRS**	**Total**	**1.33 ± 2.04**	**3.17 ± 1.64**		**Treatment group**	**13.52**	**<0.001**
	*Deciduous teeth*	1.00 ± 0.74	2.33 ± 0.65	1.67 ± 0.96	Teeth type	4.171	**0.046**
	*Permanent*	1.56 ± 2.57	3.72 ± 1.87	2.64 ± 2.48	Teeth type × Group	0.766	**0.385**
**FBRS**	**Total**	**0.47 ± 0.57**	**1.27 ± 0.45**		**Treatment group**	**31.66**	**<0.001**
	*Deciduous teeth*	0.58 ± 0.52	1.17 ± 0.39	0.88 ± 0.54	Teeth type	0.010	**0.919**
	*Permanent*	0.39 ± 0.61	1.33 ± 0.49	0.86 ± 0.72	Teeth type × Group	1.769	**0.189**
**Questionnaire**	**Total**	**8.37 ± 1.61**	**5.27 ± 1.41**		**Treatment group**	**62.378**	**<0.001**
	*Deciduous teeth*	8.50 ± 0.90	6.25 ± 1.14	7.38 ± 2.46	Teeth type	6.172	**0.016**
	*Permanent*	8.28 ± 2.96	4.61 ± 1.20	6.44 ± 2.45	Teeth type × Group	3.576	**0.064**

Notes: Data are presented as mean ± standard deviation; VAS—visual analog scale; W–BFRS—Wong–Baker Pain Rating Scale; FBRS—Frank Behavior Rating Scale; Significant differences are bolded.

## Data Availability

The original contributions presented in the study are included in the article and Supplementary Material; further inquiries can be directed to the corresponding author.

## Supplementary Materials

The following supporting information can be downloaded at https://www.mdpi.com/article/10.3390/children12020215/s1, Table S1: Structure of participants in the articaine and lidocaine group concerning gender, age, and perioperative parameters.
