# Supplementary material for: Comparative Evaluation of Alveolar Nerve Block with 2% Lidocaine–Epinephrine and 4% Articaine–Epinephrine Buccal Infiltration in Mandibular Premolar and Molar Region in Children: A Double-Blind, Randomized Trial
Source: Children (Basel). 2025 Feb 12;12(2):215. doi: 10.3390/children12020215 (PMC11854341; doi:10.3390/children12020215)
Supplement: Supplementary file 1 [file children-12-00215-s001.zip › children-3363236-supplementary.pdf]

Supplementary

Table S1. Structure of participants in the articaine and lidocaine group concerning gender, age, and perioperative parameters.

ID	Gender	Age	Group	CTR	EDT	TE	ID	Gender	Age	Group	CTR	EDT	TE
1	M	7	Artic			36	31	m	7	lidoc			36
2	F	11	Artic		36	74,75	32	f	11	lidoc		46	84,85
3	F	14	Artic			44,45	33	f	13	lidoc			34,35
4	M	9	Artic			74	34	m	9	lidoc			84
5	M	13	Artic			35	35	f	13	lidoc			45
6	F	10	Artic			75	36	f	10	lidoc			75
7	M	13	Artic			35	37	m	13	lidoc			35
8	M	14	Artic			45	38	m	14	lidoc			44
9	F	15	Artic		34		39	f	5	lidoc		35	
10	F	10	Artic	36		34,35	40	f	10	lidoc	46		35,34
11	M	14	Artic	37,36,46,47			41	m	14	lidoc	36,37,46,47		
12	F	5	Artic		75		42	f	5	lidoc		85	
13	M	11	Artic			85	43	m	10	lidoc			85,84
14	M	9	Artic	36			44	m	9	lidoc	46		
15	M	8	Artic			74,75	45	m	8	lidoc			74,75
16	F	5	Artic			74,75	46	f	5	lidoc			84,85
17	F	13	Artic	36			47	f	13	lidoc	36		
18	M	12	Artic	46			48	m	12	lidoc	46		
19	M	17	Artic	34	35		49	f	17	lidoc	44	45	
20	M	16	Artic	46			50	m	16	lidoc	46		
21	M	17	Artic	44,45			51	m	17	lidoc	44,45		
22	M	15	Artic	35,36			52	m	15	lidoc	45,46		
23	M	17	Artic		36		53	m	17	lidoc		46	
24	F	9	Artic			74,75	54	f	9	lidoc			84,85
25	F	15	Artic		46		55	f	13	lidoc		46	
26	M	8	Artic	84,85			56	m	8	lidoc	84,85		
27	F	5	Artic		85		57	f	5	lidoc		85	
28	F	6	Artic	85	84		58	f	6	lidoc	75	74	
29	F	9	Artic			74	59	f	9	lidoc			85
30	M	8	Artic			85	60	m	8	lidoc			85
